# Influence of rigid coregistration of PET and CT data on metabolic volumetry: a user’s perspective

**DOI:** 10.1186/2191-219X-3-85

**Published:** 2013-12-27

**Authors:** Ingo G Steffen, Frank Hofheinz, Julian MM Rogasch, Christian Furth, Holger Amthauer, Juri Ruf

**Affiliations:** 1 Klinik für Radiologie und Nuklearmedizin, Universitätsklinikum Magdeburg A.ö.R, Leipziger Strasse 44, Magdeburg 39120, Germany; 2 Klinik für Nuklearmedizin, Charité Centrum 6 für diagnostische und interventionelle Radiologie und Nuklearmedizin, Campus Virchow-Klinikum, Charité - Universitätsmedizin Berlin, Berlin 13353, Germany; 3 Institute of Radiopharmaceutical Cancer Research, Helmholtz-Center Dresden-Rossendorf, Dresden 01328, Germany; 4 Klinik für Nuklearmedizin, Universitätsklinikum Freiburg, Freiburg 79106, Germany

**Keywords:** Coregistration, Interpolation, PET, CT, Metabolic tumor volume, SUV

## Abstract

**Background:**

While non-rigid fusion is by definition expected to alter the information of positron emission tomography (PET) data, we assessed whether rigid transformation also influences metabolic tumor volume (MTV) determination.

**Methods:**

The PET/computed tomography (CT) data of 28 solid pulmonary lesions of 20 tumor patients examined with ^18^ F-Fluordeoxyglucose (FDG) was retrospectively analyzed. The original (OR) hardware-coregistered PET images were fused with contrast-enhanced diagnostic CT (CT1, 1 mm slices) and low dose CT (CT5, 5 mm slices). After automatic rigid transformation (Mirada Fusion7D) using two algorithms (rigid fast (RF), rigid slow (RS)), MTV and maximal standardized uptake value (SUVmax) were determined applying four different segmentation methods with either fixed or background-adapted thresholding and compared to OR-PET data.

**Results:**

Relative differences in SUVmax compared to OR data revealed no significant differences for RF (median, −0.1%; interquartile range (IQR), −1.1% to 0.9%; *p* = 0.75) and RS (median, 0.5%; IQR, −0.6% to 1.3%; *p* = 0.19) in CT1, whereas in CT5 significant deviations were observed for RF (median, −9.0%; IQR, −10.9 to −6.1; *p* < 0.001) and RS (median, −8.4%; IQR, −11.1 to −5.6; *p* < 0.001). Relative MTV differences were 0.7% (IQR, −3.0% to 2.7%; *p* = 0.76) for RF and −1.3% (IQR, −3.6% to 0.9%; *p* = 0.12) for RS in CT1. Coregistration led to significant MTV differences in RF (median, 10.4%; IQR, 7.4% to 16.7%; *p* < 0.001) and RS (median, 10.6%; IQR, 5.4% to 17.7%; *p* < 0.001) in CT5.

**Conclusions:**

Rigid coregistration of PET data allows a quantitative evaluation with reasonable accuracy in most cases. However, in some cases, it can result in substantial deviations of MTV and SUVmax. Therefore, it is recommended to perform quantitative evaluation in the original PET data rather than in coregistered PET data.

## Background

Image fusion, initially software-based, is an established procedure in nuclear medicine (NM) [1,2], and both rigid and non-rigid coregistration approaches still are the research focus of many groups [3,4]. While modern hybrid-tomographs usually provide an appropriate alignment of functional positron emission tomography (PET) and morphological computed tomography (CT), changes in breathing pattern between both examinations or physiological organ and/or patient movement may lead from slight to grave incongruences of the two 3D-data sets to be matched [5-8]. Thus, automatic or manual software-based fine-tuning is commonplace in order to create a better match of the relevant anato-metabolic findings [9].

In PET imaging, it is commonplace to assess tumor metabolism not only qualitatively (i.e., visual analysis) but also quantitatively. Apart from the established standardized uptake value (SUV) determination, more recently tumor volume measurements have been reported that could be of value especially in therapy assessment [10] and radiation oncology [11-13]. In addition to intrinsic hardware-based image fusion supplied by PET/CT-hybrid-devices, software-based fine-tuning is possible for correction of fusion artifacts or is even necessary, for example, for the planning of radiation therapy [11].

While elastic (non-rigid, deformable) fusion is expected to alter the information of PET data, the present study assessed whether the corresponding interpolation also has an impact on semiquantification in a rigid (translations and/or rotations only) transformation setting.

## Methods

### Patients

The PET/CT data of 20 lung cancer patients (12 male, 8 female; median age, 71.3 years; range, 57 to 82 years) with a total of 28 solid lung lesions were included. This retrospective study was approved by the local ethics committee (application no. EA2/143/12), and all patients signed a written informed consent.

### PET/CT data acquisition

Patients received a whole-body PET/CT examination with ^18^ F-Fluordeoxyglucose (FDG) (Biograph 16, Siemens Medical, Erlangen, Germany). The PET protocol included an 8-h fasting period followed by confirmation of a blood glucose level ≤ 110 mg/dl prior to the scanning procedure. PET scans were performed 90 min after intravenous injection of 250 to 380 MBq (median, 300 MBq) FDG (five to six bed positions at 3 min each; matrix size, 168 × 168; voxel size, 4.1 × 4.1 × 5.0 mm).

CT imaging first consisted of an unenhanced low-dose CT (40 mAs/120 kV; detector collimation, 16 × 1.5 mm) reconstructed with a slice thickness of 5 mm (matrix size, 512 × 512; voxel size, 1.4 × 1.4 × 5.0 mm). From this scan also, the attenuation map necessary for the attenuation correction of PET data was derived. Subsequently, a dedicated CT scan using CareDose4D® (part of Somatom 16 Software, Version B10, Siemens Medical, Erlangen, Germany) automatic dose regulation technology (230 mAs/120 kV; detector collimation, 16 × 1.5 mm) was performed using intravenous contrast enhancement (70 to 100 ml Ultravist 370, Bayer Schering Pharma, Berlin, Germany/venous phase with 70 s delay) and a reconstructed slice thickness of 1 mm (matrix size, 512 × 512, voxel size, 1.4 × 1.4 × 1.0 mm).

For all scans, the patients were in the supine position with arms elevated and they were instructed to retain a shallow breathing pattern throughout the low-dose CT scan and the PET acquisition in order to minimize motion-induced attenuation correction artifacts. The diagnostic CT scan was acquired during the inspiration phase. PET images were reconstructed using an iterative two-dimensional ordered subset expectation maximization algorithm (2D OSEM, 4 iterations, 8 subsets, 5 mm FWHM Gaussian filter) including correction for scatter and attenuation.

### Image registration

Coregistration was performed using Mirada Fusion 7D (Build FUSM 1.0.0.8, Broker 5.5.6.7, Mirada Solutions, Oxford, UK) on a Leonardo workstation (CPU, Intel Xeon 3,2 GHz; OS, Windows XP Prof., SP3; RAM, 3GB; e.soft Software, Version 4.0, Siemens Medical Solutions, Erlangen, Germany). Only thoracic slices (lung apices to diaphragm) were selected for coregistration. Digital imaging and communications in medicine (DICOM) images were transferred from the workstation to the Mirada software using the Mirada DICOM broker selecting CT images as source data and corresponding PET images as target data. Rigid coregistration was performed using the ‘rigid fast’ (RF) and the ‘rigid slow’ (RS) mode available in the Mirada software. Both algorithms are based on mutual information with the main difference being the number of samples used for the similarity function (for example, the slow algorithm is supposed to be more accurate but is more time-consuming). Original PET data as well as coregistered PET data were saved in the CT geometry and pixel size for CT1 and CT5, respectively. In the following, we refer to the resampled original data (hardware coregistered) as original (OR) data.

The median coregistration times for CT5 were 7.4 s (interquartile range (IQR), 6.7 to 7.9 s) using RF and 101.2 s (IQR, 92.5 to 119.3 s) applying RS with a corresponding median ratio of 15.6 (IQR, 13.1 to 16.5). In CT1, the median coregistration times were 9.1 s (IQR, 8.3 to 10.2 s) for RF and 80.6 s (IQR, 73.2 to 92.5 s) for RS with a corresponding median ratio of 8.6 (IQR, 8.0 to 10.1).

### Metabolic volumetry

Lesions in PET were delineated using dedicated software (Rover, Version 2.1.8, ABX GmbH, Radeberg, Germany) applying four different segmentation methods. First, an adaptive threshold method (AT), which applies a volume-reproducing threshold after subtraction of local background, was used [14]. The other segmentation methods are based on fixed thresholds delineating all voxels with an activity concentration of at least 40% (T40), 50% (T50), or 60% (T60) of the measured maximum activity, respectively. In all lesions, SUVmax, metabolic tumor volume (MTV) and total lesion glycolysis (TLG, MTV*SUVmean) were determined for OR and for the coregistered (RS/RF) PET data, using both CT1 and CT5.

### Statistical methods

All calculations were performed using the R-system for statistical computing (version 2.15.3, R Foundation for statistical Computing, Vienna, Austria, http://www.R-project.org). Descriptive parameters were expressed as mean, median, IQR, and range. Differences between original and coregistered date were analyzed using nonparametric Wilcoxon test for paired data. Agreement of different methods was analyzed using Bland-Altman plots [15] and 95% limits of agreement (95% LoA). All tests were two-sided, and statistical significance was assumed at *p* < 0.05.

## Results and discussion

### Results

#### Original PET data

The MTV of original PET data was 3.2 (IQR, 2.4 to 6.2) ml and ranged from 1.1 to 27.2 ml with a median SUVmax of 9.0 (5.6 to 12.8) ranging from 1.6 to 30.9. The TLG of original PET data showed a median of 13.9 (8.7 to 51.7) ml and ranged from 3.4 to 370.1 ml. After the resampling step leading to OR data, the SUVmax remained essentially unchanged (median, 8.8; IQR, 5.8 to 12.6; range, 1.6 to 30.3).

#### SUVmax differences between OR and coregistered PET data

*CT1.* Median SUVmax in OR was 8.8 (5.6 to 12.6) and showed no significant differences compared to median SUVmax of 8.7 (5.6 to 12.5) in RF (*p* = 0.75) and 8.7 (5.6 to 12.5) in RS (*p* = 0.19). The median relative SUVmax differences were −0.1 (−1.1% to 0.9%) ranging between −2.3% and 2.8% in RF and 0.5 (−0.6% to 1.3%) ranging between −2.3% and 2.5% in RS.

*CT5.* Median SUVmax decreased significantly from 8.8 (5.6 to 12.6) in OR to 8.1 (5.2 to 11.7) in RF (*p* < 0.001) and to 8.0 (5.4 to 11.5) in RS (*p* < 0.001) with median relative SUVmax differences of −9.0 (−10.9% to −6.1%) ranging from −18.2% to −1.1% in RF and with median relative SUVmax differences of −8.4 (−11.1% to −5.6%) ranging from −19.4% to 4.3% in RS.

#### MTV differences between OR and coregistered PET data

*CT1.* Median MTV in OR was 3.3 (2.4 to 5.9) ml and showed no significant differences compared to median MTV of 3.1 (2.3 to 5.9) ml in RF (*p* = 0.76) and 3.1 (2.3 to 5.8) ml in RS (*p* = 0.12). Relative MTV differences ranged from −12.7% to 9.2% (median, 0.7%; IQR, −3.0% to 2.7%) for RF and from −16.1% to 14.1% (median, −1.3%; IQR, −3.6% to 0.9%) for RS.

*CT5.* While the median MTV in OR measured 3.3 ml (IQR, 2.4 ml to 6.0 ml), it increased significantly to 3.6 ml (IQR, 2.7 to 6.4 ml) in RF (*p* < 0.001) and to 3.5 ml (IQR, 2.7 to 6.5 ml) in RS (*p* < 0.001). Accordingly, relative MTV differences showed a median of 10.4% (IQR, 7.4% to 16.7%; range, −11.7% to 48.0%) in RF and 10.6% (IQR, 5.4% to 17.7%; range, −17.0% to 42.7%) in RS.

#### TLG differences between OR and coregistered PET data

*CT1.* OR data featured a median TLG of 12.5 (8.1 to 48.4) ml with no significant differences compared to 12.7 (8.4 to 47.8) ml in RF (*p* = 0.40) and 12.5 (8.1 to 47.5) ml in RS (*p* = 0.12). The corresponding median relative TLG differences were 0.5 (−2.2% to 1.9%) ranging from −9.9% to 6.5% in RF and −0.9 (−2.4% to 0.6%) ranging from −12.6% to 10.2% in RS.

*CT5.* The median TLG in OR was 13.6 (8.5 to 50.5) ml and increased significantly to 13.7 (9.8 to 51.0) ml in RF (*p* < 0.05). In contrast, no significant difference was observed after coregistration with RS with a median of 14.4 (9.2 to 51.1) ml (*p* = 0.13). The median relative TLG differences measured 2.0 (−0.5% to 4.8%) ranging between −12.2% and 22.1% in RF and 1.6 (−2.1% to 5.8%) ranging between −15.7% and 20.8% in RS.

#### Association of MTV and segmentation methods

*CT1.* Relative MTV differences for RF based on different segmentation algorithms showed a median of 0.7 (IQR, −3.0% to 2.7%) (AT), 0.2 (−1.2% to 1.8%) (T40), 0.3 (−1.8% to 2.7%) (T50), and 0.7 (−2.6% to 4.0%) (T60), respectively. The corresponding results for RS were −1.3 (−3.6% to 0.9%) (AT), −1.0 (−2.8% to 1.2%) (T40), −1.0 (−2.5% to 1.4%) (T50), and −1.3 (−4.0% to 1.2%) (T60). For RF as well as RS data, the Wilcoxon test revealed significant differences neither between AT and T40 (RF, *p* = 0.69; RS, *p* = 0.35), nor between AT and T50 (RF, *p* = 0.49; RS, *p* = 0.35), and nor between AT and T60 (RF, *p* = 0.49; RS, *p* = 0.70).

*CT5.* Median relative MTV differences for RF measured 10.5 (7.4% to 16.7%) (AT), 12.9 (9.1% to 21.2%) (T40), 11.7 (7.8% to 17.2%) (T50), and 9.4 (4.8% to 15.4%) (T60), respectively. Relative MTV differences for RS showed a median of 10.6 (5.4% to 17.7%) (AT), 12.6 (8.1% to 23.4%) (T40), 13.1 (7.4% to 18.6%) (T50), and 9.9 (3.4% to 17.8%) (T60). For RF as well as RS data, the Wilcoxon test revealed significant differences between AT and T40 (both *p* < 0.05) but not between AT and T50 (RF, *p* = 0.20; RS, *p* = 0.12) or AT and T60 (RF, *p* = 0.69; RS, *p* = 0.45), respectively.

**Figure 1:**
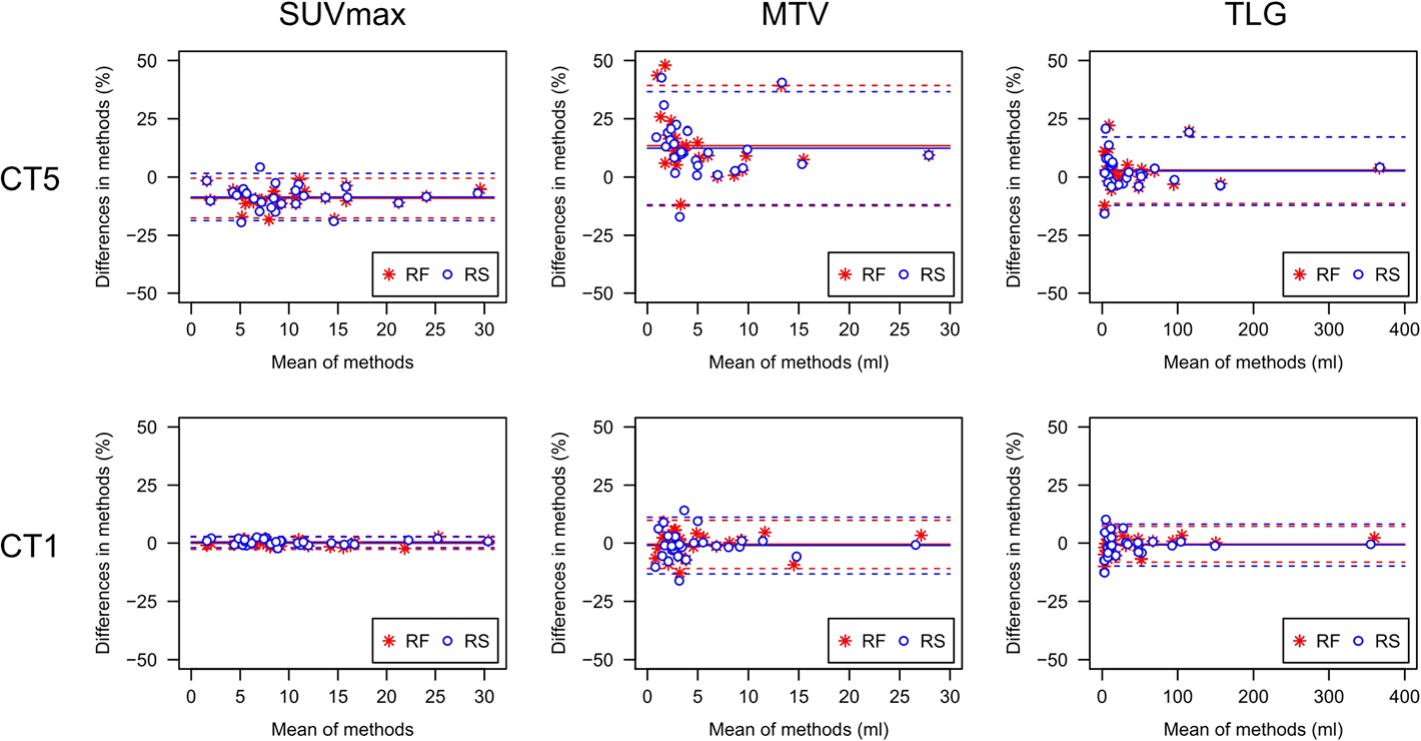
**Bland-Altman plots of relative changes in SUVmax, MTV, and TLG.** Relative changes after coregistration to CT1 and CT5 using the RF and RS coregistration algorithm of 28 lesions in 20 patients. Differences are calculated as coregistered data - original data. Solid and dashed lines represent mean ± 2SD.

**Figure 2:**
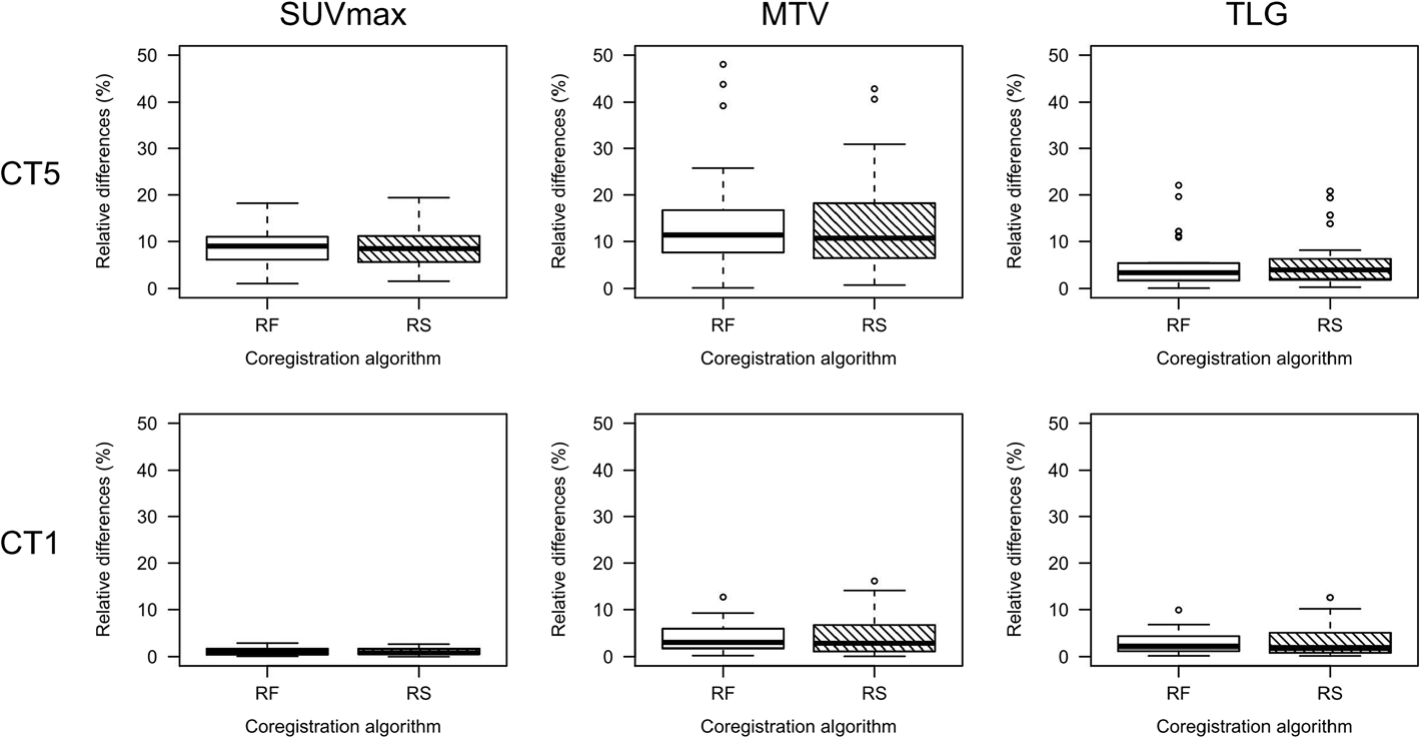
**Box plots of relative changes in SUVmax, MTV, and TLG.** Relative changes for CT5 and CT1 using the RF and RS coregistration algorithm of 28 lesions in 20 patients (unsigned). Outliers are marked by circles.

**Figure 3:**
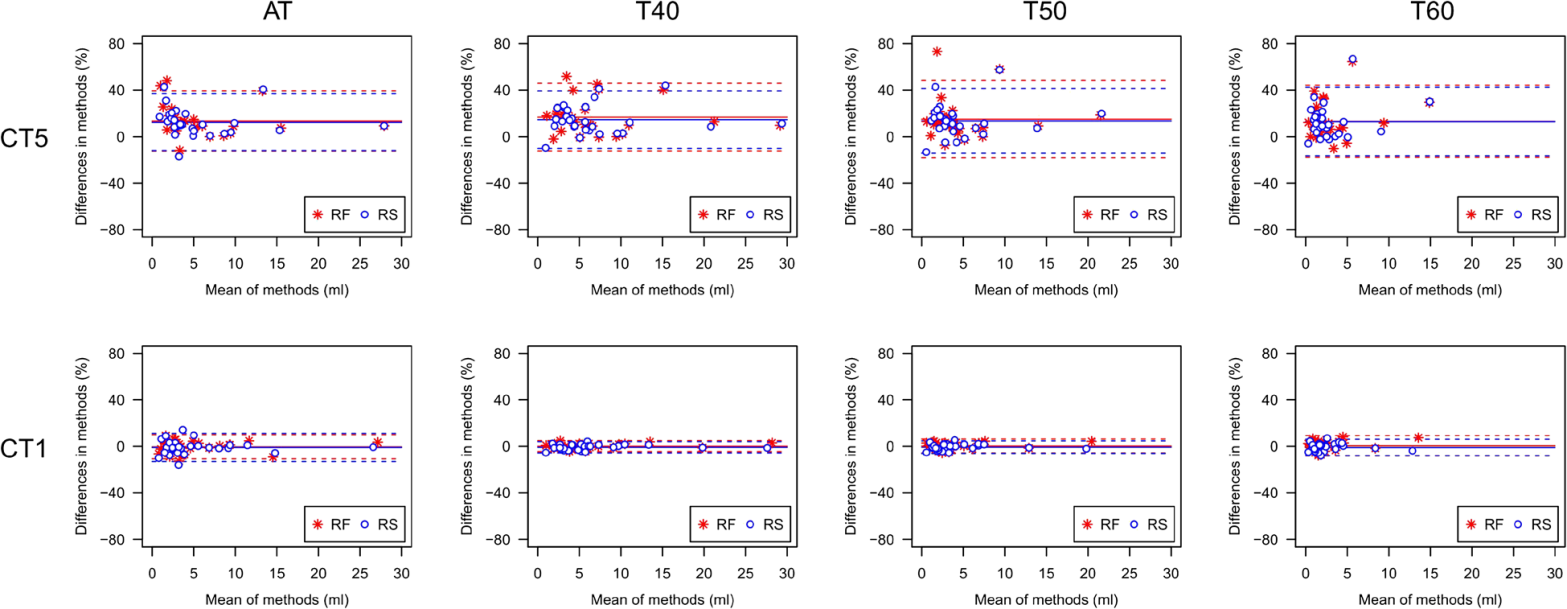
**Bland-Altman plots of relative changes in MTV for different segmentation algorithms.** Relative MTV changes for different segmentation algorithms after coregistration to CT1 and CT5 using the RF and RS coregistration algorithm in 28 lesions in 20 patients. Differences are calculated as coregistered data - original data. Solid and dashed lines represent mean ± 2SD.

**Figure 4:**
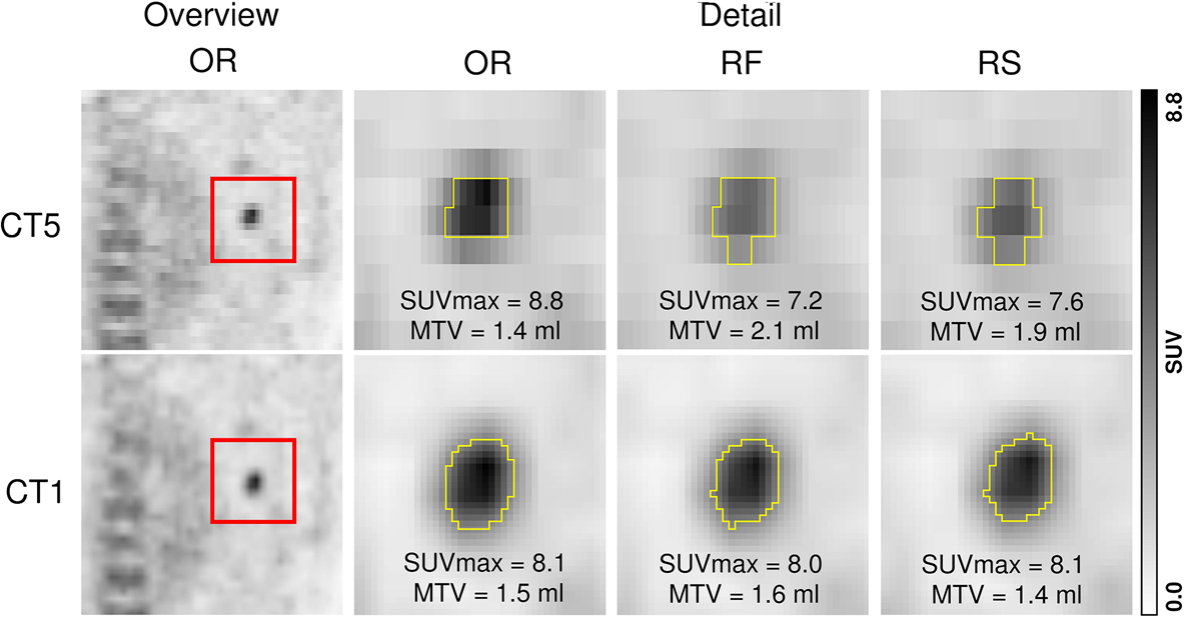
**Coronal visualization of a pulmonary PET positive lesion coregistered to CT1 and CT5.** The anatomical localization is indicated by the red square. The detailed view of this region shows the respective delineation (yellow line) of the voxels included in the MTV-calculation of the original scan (OR) and after transformation (RF, RS).

**Figure 5:**
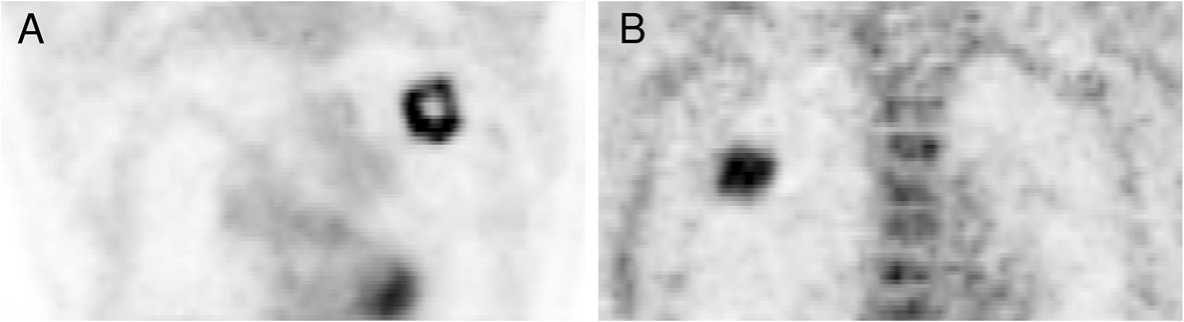
**Coronal visualization of two pulmonary PET positive lesions.** The lung lesion exhibiting approximately the same MTV but have different heterogeneity. The first lesion **(A)** has a central necrosis, leading to relatively high differences (SUVmax, 17.9%; MTV, 39.2%; TLG, 19.6%) after coregistration. For the second, rather homogeneous lesion **(B)**, the differences are substantially lower (SUVmax, 7.4%; MTV, 7.6%; TLG, 1.4%).

Descriptive parameters of unsigned absolute and relative differences between OR and coregistered PET data are given in Tables 1, 2, and 3. Corresponding relative differences are depicted as Bland-Altman plots in Figure 1. The unsigned relative differences are displayed as boxplots in Figure 2. Relative MTV differences between OR and coregistered PET data for different segmentation algorithms are presented in Table 4 and depicted as Bland-Altman plots in Figure 3. An example demonstrating the differences of MTV and SUVmax between OR and coregistered PET data is given in Figure 4. Figure 5 illustrates the influence of tracer distribution on interpolation effects, e.g., due to tumor heterogeneity.

**Table 1:** Unsigned differences in SUVmax after coregistration

	**RF**		**RS**	
	**∆SUVmax**	**∆SUVmax (%)**	**∆SUVmax**	**∆SUVmax (%)**
***CT1***				
Mean	0.1	1.1	0.1	1.0
Median	0.1	1.0	0.1	0.8
IQR	0 to 0.2	0.4 to 1.6	0.0 to 0.1	0.5 to 1.5
Range	0.0 to 0.7	0.1 to 2.8	0.0 to 0.5	0.0 to 2.5
***CT5***				
Mean	0.9	9.0	1.0	8.8
Median	0.7	9.0	0.8	8.4
IQR	0.5 to 1.3	6.1 to 10.9	0.3 to 1.3	5.6 to 11.1
Range	0.0 to 2.9	1.1 to 18.2	0.0 to 3.0	1.6 to 19.4

RF, rigid fast; RS, rigid slow; CT1, 1-mm-slice thickness; CT5, 5-mm-slice thickness.

**Table 2:** Unsigned differences in metabolic tumor volumes (MTV) after coregistration

	**RF**		**RS**	
	**∆MTV (ml)**	**∆MTV (%)**	**∆MTV (ml)**	**∆MTV (%)**
***CT1***				
Mean	0.2	4.0	0.2	4.4
Median	0.1	2.9	0.1	2.7
IQR	0.1 to 0.2	1.8 to 5.8	0.1 to 0.2	1.2 to 6.4
Range	0.0 to 1.4	0.2 to 12.7	0.0 to 0.9	0.0 to 16.1
***CT5***				
Mean	0.6	14.4	0.6	13.7
Median	0.4	11.4	0.4	10.7
IQR	0.3 to 0.5	7.6 to 16.7	0.2 to 0.6	6.8 to 17.7
Range	0.0 to 4.3	0.1 to 48.0	0.0 to 4.5	0.8 to 42.7

RF, rigid fast; RS, rigid slow; CT1, 1-mm-slice thickness; CT5, 5-mm-slice thickness.

**Table 3:** Unsigned differences in TLG after coregistration

	**RF**		**RS**	
	**∆TLG (ml)**	**∆TLG (%)**	**∆TLG (ml)**	**∆TLG (%)**
***CT1***				
Mean	0.9	2.9	0.6	3.2
Median	0.4	2.1	0.4	1.8
IQR	0.2 to 0.7	1.2 to 4.2	0.2 to 0.8	0.8 to 4.8
Range	0.0 to 8.1	0.2 to 9.9	0.1 to 2.2	0.2 to 12.6
***CT5***				
Mean	2.1	5.3	2.1	5.4
Median	0.6	3.2	0.7	3.8
IQR	0.2 to 1.8	1.7 to 5.3	0.5 to 0.9	1.9 to 6.1
Range	0.0 to 20.6	0.1 to 22.1	0.0 to 20.3	0.3 to 20.8

RF, rigid fast; RS, rigid slow; CT1, 1-mm-slice thickness; CT5, 5-mm-slice thickness.

**Table 4:** Relative differences in MTV for different segmentation algorithms (AT, T40, T50, T60) after coregistration

	**AT**	**T40**	**T50**	**T60**
***CT1 (RF)***				
Mean	−0.5	0.1	0.2	0.5
Median	0.7	0.2	0.3	0.7
IQR	−3.0 to 2.7	−1.2 to 1.8	−1.8 to 2.7	−2.6 to 4.0
Range	−12.7 to 9.2	−4.7 to 4.7	−5.8 to 4.3	−7.9 to 8.1
***CT5 (RF)***				
Mean	13.6	16.9	15.1	13.2
Median	10.5	12.9	11.7	9.4
IQR	7.4 to 16.7	9.1 to 21.2	7.8 to 17.2	4.8 to 15.4
Range	−11.7 to 48.0	−1.9 to 51.6	−7.1 to 73.1	−10.0 to 64.5
***CT1 (RS)***				
Mean	−1.0	−0.9	−0.8	−1.1
Median	−1.3	−1.0	−1.0	−1.3
IQR	−3.6 to 0.9	−2.8 to 1.2	−2.5 to 1.4	−4.0 to 1.2
Range	−16.1 to 14.1	−5.5 to 4.1	−5.6 to 5.3	−8.0 to 6.9
***CT5 (RS)***				
Mean	12.5	14.7	13.6	13.0
Median	10.6	12.6	13.1	9.9
IQR	5.4 to 17.7	8.1 to 23.4	7.4 to 18.6	3.4 to 17.8
Range	−17.0 to 42.7	−9.5 to 44.0	−13.2 to 57.4	−5.8 to 66.8

RF, rigid fast; RS, rigid slow; CT1, 1-mm-slice thickness; CT5, 5-mm-slice thickness.

### Discussion

Recent uses of PET data for staging, therapy assessment, and definition of target volume for irradiation indicate that the sole definition of the traditional SUVmax, based on a single voxel, may be insufficient. As a consequence, the assessment of the whole metabolic tumor volume or the determination of total lesion glycolysis [16] has been suggested. However, the accurate delineation of a PET positive lesion is difficult as manual segmentation is associated with a large intra- and interobserver variability and fixed thresholds have been proven to be inadequate [17]. To overcome this problem, several automatic delineation methods have been proposed [18-23], but up to the present, no general consensus about the best method exists. In this study, we used a method which applies a volume-reproducing intensity threshold after subtraction of local background. The method seems promising [14] and is implemented in commercial software available at our site. Since fixed thresholds are still frequently used [24,25], we also investigated three different thresholds (see the following paragraphs).

It has already been shown that quantification of PET is affected by a multitude of biological and technical factors influencing PET acquisition and reconstruction [26,27]. The effect of different reconstruction algorithms on PET-based volume segmentation was analyzed in a recent study and showed a substantial influence of reconstruction algorithms on segmentation thresholds [28]. In the present study, the images were reconstructed as usually performed in our clinical routine (2D-OSEM), and the effect of rigid coregistration on PET quantification was observed. The applied two mutual information-based rigid coregistration algorithms (RF and RS) allow user-independent translations and rotations of the PET data. Both algorithms showed deviations of SUVmax and metabolic tumor volume compared to original coregistration algorithm in a similar range.

However, it has to be emphasized that the focus of the recent study was not to determine the accuracy of the different coregistration algorithms as no reference standard was available for this issue. It is obvious that the final result of the OR data may be influenced by motion blur or incongruence of PET and CT data due to different organ positions (not to mention the attenuation correction errors associated therewith) [29]. However, the aim was to demonstrate in a proof of principle that notable deviations can be observed also after mere rigid transformations using coregistration algorithms as usually performed in clinical routine.

On average, the observed deviations of SUVmax, MTV, and TLG are rather small (see Tables 1, 2, and 3). For the PET data coregistered to CT1 also, the maximum deviations were moderate (SUVmax, 2.8%; MTV, 16.1%; TLG, 12.6%) while the PET data coregistered to the coarsely sliced CT5 showed strong deviations in some cases (maximum differences, SUVmax, 19.4%; MTV, 48.0%; TLG, 22.1%). This is an expected result, since in general on a coarse grid, interpolation effects are more pronounced than on a fine grid independent of the interpolation method. CT5 had a slice thickness of 5 mm compared to 1 mm of CT1. The in-plane voxel size was the same for both CTs. It can be expected that for larger in-plane voxel sizes (e.g., coregistration of two follow up PETs), deviations are even larger and occur more often.

Besides the target voxel grid, the interpolation method directly influences the deviation of SUVmax, MTV, and TLG. The interpolation method implemented in the used coregistration software is trilinear interpolation. Therefore, our results are strictly speaking only valid for the applied coregistration software. However, similar effects can be expected with other coregistration software.

The transformation parameters used for coregistration also have direct influence on the interpolation effects. If, for example, the data are shifted by a multiple of the voxel size, the data are not interpolated at all. On the other hand, if the data are shifted by half of the voxel size (plus an arbitrary multiple of the voxel size), the interpolation effects are maximal. This effect can also be observed in our results: all large deviations in SUVmax, MTV, and TLG were associated with shifts clearly deviating from multiples of the voxel size while in most other cases, the shifts were close to a multiple of the voxel size. In general, the transformation parameters, necessary for an optimal coregistration, are not predictable and, thus, also the magnitude of the interpolation effects is not predictable.

Finally, the immediate neighborhood of the maximum voxel determines the magnitude of the potential interpolation effect. For a large homogeneous lesion, the difference of maximum voxel and neighboring voxel is just noise and the interpolation effects essentially lead to noise reduction (assuming trilinear interpolation). This is different for small or heterogeneous lesions. For small lesions (compared to the spatial resolution), the neighborhood of the maximum voxel is usually lowered by partial volume effects, and therefore, interpolation effects are increased. It should be noted that for very small lesions, the maximum voxel itself is compromised by limited signal recovery already in the original image and should be interpreted with care even without interpolation effects.

However, also larger but heterogeneous lesions can have substantial interpolation effects as can be seen in Figure 5, where two lesions with approximately the same volume but different heterogeneity are shown. For the heterogeneous lesion (Figure 5A), the SUVmax is decreased by 18%, MTV is increased by 39%, and TLG by 20% (all for CT5 and RF) which might be a notable impact, for example, on the repeated measurement in follow-up studies due to error propagation or in the field of PET-based planning of radiotherapy [30,31] where an accurate MTV definition is important. On the other hand, for the rather homogeneous lesion shown in Figure 5B, the deviation of SUVmax and MTV is approximately 8% (TLG even lower) which is acceptable in most cases.

MTV was determined with an adaptive threshold method, which is routinely used at our site. Additionally, the lesions were delineated applying three different fixed thresholds. For all delineation methods, the deviation of MTV was comparable, where the adaptive threshold method resulted in slightly lower deviations for CT5 and the fixed threshold method showed slightly lower deviations for CT1 (see Figure 3). It should be noted that the present study assessed only the deviation of MTV after coregistration but not the delineation accuracy.

A recent study analyzed the effect of rigid and non-rigid coregistration methods on SUVmax and MTV in association with different breathing maneuvers in patients with lung cancer [4]. Whereas MTV was significantly influenced by the choice of registration method depending on breathing protocol, no significant impact on SUVmax was observed. As the present study analyses the difference between coregistered and original data, a comparison of both studies is difficult. However, the reported range of mean relative SUVmax changes (−18% to 20%) and MTV changes (−43% to 61%) corresponds to the current study. A further study investigated the effect of rigid and non-rigid image registration on test-retest (TRT) variability of SUV and MTV in patients with colorectal carcinoma [3]. Significant differences in TRT compared to the reference were only observed for the MTV after rigid registration but neither for MTV after non-rigid registration nor for SUVmax. As this study is based on two different scans for each patient, the design differs substantially from the current approach. However, median TRT variability was about 10% for SUVmax and 15% for MTV in the reference group with corresponding maximums of about 25% for SUVmax and 50% for MTV which is in the range of the present study.

A limitation of our study is that only threshold-based delineation methods were used for MTV determination. It cannot be excluded that the observed deviations of MTV are partly caused by using only such methods. Other delineation algorithms which are not based on thresholding [21-23] might be less sensitive to interpolation effects. However, since these algorithms are not available at our institution, the observed effects represent our clinical routine.

## Conclusions

The interpolation due to rigid coregistration of PET and CT data still allows a quantitative evaluation of PET data with a reasonable accuracy in most cases. However, in some cases, it can result in substantial deviations of MTV and SUVmax. The magnitude of the deviation depends on several factors and is in general not predictable. Therefore, it is recommended to perform quantitative evaluation in the original PET data rather than in coregistered PET data.

## Competing interests

The authors declare that they have no competing interests.

## Authors’ contributions

IS and JR participated in the design of the study and in the analysis and interpretation of data and drafted the manuscript. FH, JMMR, CF, and HA participated in the analysis and interpretation of data and supervised the manuscript. All authors read and approved the final manuscript.
